# Alternative hosts of *Leishmania infantum*: a neglected parasite in Europe

**DOI:** 10.1007/s11250-024-03978-0

**Published:** 2024-04-17

**Authors:** Ioannis Tsakmakidis, Menelaos Lefkaditis, Konstantinos Zaralis, Georgios Arsenos

**Affiliations:** 1 School of Agricultural Sciences, Department of Agriculture, University of Western Macedonia, end of Kontopoulou str, Florina, 53100 Greece; 2 Faculty of Veterinary Science, University of Thessaly, Karditsa, Greece; 3 Faculty of Veterinary Medicine, Aristotle University of Thessaloniki, Thessaloniki, Greece

**Keywords:** Domestic animals, Europe, *Leishmania Infantum*, Leishmaniosis, Tropics, Wild animals

## Abstract

Multi-host pathogens that infect various animal species and humans are considered of great importance for public and animal health. *Leishmania* spp. parasites are a characteristic example of such pathogens. Although leishmaniosis in humans is endemic for about 100 countries around the world it is classified as a neglected tropical disease. There are three main forms of leishmaniosis in humans: cutaneous (CL), visceral (VL) and mucocutaneous leishmaniosis (MCL). Each year, about 30,000 new cases of VL and more than 1 million new cases of CL are recorded. In Europe *L. infantum* is the dominant species with dogs being reservoir hosts. Apart from dogs, infection has been recorded in various animals, which suggests that other species could play a role in the maintenance of the parasite in nature. Herein we provide an in-depth review of the literature with respect to studies that deal with *Leishmania infantum* infections in domestic and wild animal species in Europe. Given the fact that domesticated and wild animals could contribute to the incidences of leishmaniosis in humans, the aim of this paper is to provide a comprehensive review which could potentially be used for the development of measures when it comes to the control of the *Leishmania infantum* parasite.

## Introduction

Pathogens that infect different animal species and humans are of great importance for public health. Among of all human pathogens, 61% are zoonotic, infecting both humans and animals (Taylor et al., 2001). A large proportion of pathogens that use specific animal species as hosts in their biological cycle can also be transmitted and infect other domestic or wild animals, influencing about 77% of livestock species and 90% of carnivores (Cleaveland et al., 2001). Given the notion that these pathogens can spread widely amongst humans or emerge constantly from their reservoirs in nature, zoonotic infections could have a major impact both in public health and the economy (Bowden and Drake, 2013). According to the World Organization for Animal Health, 75% of emerging infectious diseases in humans have their origins in domestic and wild animals (Fong, 2017). Even though many of these diseases are considered to have more than one reservoir hosts in nature, these hosts in many cases are not yet identified (Daszak et al., 2000). *Leishmania* is a genus of parasites that represents a typical example of a multi-host pathogen, important for both public and animal health (Haydon et al., 2002).

### Parasite and transmission

The genus *Leishmania* consists of dimorphic, vector-borne protozoan-parasites that belong to the order Kinetoplasida and the family Trypanosomatidae and are responsible for the mammalian parasitic disease of leishmaniosis (Dedet, 2002). The species of the genus *Leishmania* have a complex biological life cycle and use a vertebrate host, bearing the amastigote form of the parasite, that multiplies into the macrophages and into the mononuclear phagocytes and an invertebrate vector (i.e. phlebotomine sand fly) that carries the promastigote form of the parasite (CDC, 2017). Worldwide there are more than 800 known sand fly species, 78 which are proven vectors of the parasite (Killick-Kendrick, 1990; Banuls et al., 2007; Ready, 2010; Akhoundi et al., 2016). Two out of the six described sand fly genera, namely *Phlebotomus* (Old World) and *Lutzomyia* (New World) are proven vectors of *Leishmania* spp. and thus important for human and animal health (Killick-Kendrick, 1999; Zavitsanou et al., 2008). Transmission in nature among the parasite’s vertebrate hosts occurs in most cases by the bite of the female infected sand fly (order Diptera, family Psychodidae; subfamily Phlebotominae) (Ready, 2014; Maroli et al. 2013). Apart from this, there are some exceptional ways of transmission. In humans, the parasites can be transmitted congenitally, sexually, by blood transfusion, by transplants and finally by the sharing of infected needles, a fact that could explain the large number of *Leishmania*/HIV co-infections (Desjeux, 1999; W.H.O., 2000; Pagliano et al., 2005; Boehme et al., 2006). In dogs, it is believed that transmission can take place by biting, by blood transfusion, sexually and congenitally, with the last two also proven experimentally (Rosypal et al., 2005; Quinnell and Courtenay, 2009; Silva et al., 2009; CFSPH, 2022).

### Leishmania species

Globally, there are about 30 species of *Leishmania* parasites that are transmitted to mammals. These species are divided into 2 subgenera: (1) *Leishmania*, in which the parasites develop in the midgut and foregut of the vector; and (2) *Viannia*, in which the parasites undergo further development in the hindgut (Desjeux, 1996). At least 20 of these species are transmitted and can cause disease to humans. Humans are the primary reservoir host for two of these species, i.e., *Leishmania donovani* and *Leishmania. tropica* that are recognized as strictly anthroponotic, while the rest of the species are considered zoonotic (Desjeux, 2004; WHO, 2010). Most of the animals do not show any obvious sign of disease, in many cases the parasitic load is very low, and the host response is minimal or cannot be detected. The dog represents an exception to this general rule, as it is a very susceptible animal to the infection. Dogs are considered to be the main reservoir host of *L. infantum* in nature, and suffer from a severe and fatal disease, canine leishmaniosis (CanL) (WHO, 2010; Gramiccia, 2011). In humans, leishmaniosis (HumL) has three main clinical forms: cutaneous leishmaniosis-CL (localized or diffuse), mucocutaneous leishmaniosis-ML and visceral leishmaniosis-VL. This variability of clinical features is due to the diversity of *Leishmania* spp. and the immune response of the hosts. On the other hand, infected people can have a silent infection, without any development of symptoms (Ready, 2010; CDC, 2020).

### Disease prevalence

Leishmaniosis is endemic in around 100 countries in tropical, subtropical and temperate territories of the world (Alvar et al., 2012; Faiman et al., 2013). Human population that could encounter aa risk in these endemic areas are estimated to be more than one billion (Alvar et al., 2006; Torres-Guerrero et al., 2017). The disease is the third most important vector-borne disease after malaria and lymphatic filariosis (Pennisi, 2015) and although leishmaniosis is estimated to be the cause of the ninth largest burden among individual infectious diseases, it is still one of the world’s most neglected and underreported disease (WHO, 2010; Gramiccia, 2011). Each year, about 30 000 new cases of VL and more than 1 million new cases of CL occur in the endemic zones of the word i.e. Latin America, Africa, India, Middle East and Mediterranean region. The occurrence of leishmaniosis is not uniformly distributed: 90% of VL clinical cases occur in only six countries (i.e. India, Bangladesh, Sudan, South Sudan, Ethiopia and Brazil) (Alvar et al., 2012; Gradoni, 2013). On the other hand, CL is more widely distributed, and 90% of the cases occur every year in Afghanistan, Algeria, Iran, Saudi Arabia, Syria, Bolivia, Brazil, Colombia, Nicaragua and Peru (WHO, 2010; Rezvan, 2014). In the Mediterranean area, four *Leishmania* spp. are present: (1) *L. infantum*, is the most common one and the causative pathogen for the human VL and CL and the CanL of dogs, (2) *L. major* causes CL in the North Africa and the Middle East, (3). *L. tropica* causes CL in Greece, Turkey, the Middle East and North Africa and finally, (4) *L. donovani*, which is a recently reported species in Cyprus and can cause both VL and CL (Antoniou et al., 2013). Leishmaniosis is endemic in all of the southern European countries and is the only tropical vector-borne disease that has been endemic in that area for decades (Dujardin et al., 2008). There are two major epidemiological entities in Europe: (1) zoonotic VL and CL due to *L. infantum* infection, being present in all the southern European countries, where dogs are the main reservoir hosts of the parasite, and (2) anthroponotic CL, due to *L. tropica* infection, occurring sporadically in Greece, with most reported cases being of zoonotic visceral leishmaniοsis (ZVL) (Gradoni, 2013). In the endemic areas of Europe reported clinical cases of HumL range between 0.02 and 0.49/100,000; this means that annually about 700 new clinical cases occur in Europe (Dujardin et al., 2008; Di Muccio et al., 2015). It should be noted that there are many human carriers of the parasite (Michel et al., 2011). In addition, it is estimated that for each reported clinical case of VL there are in addition 30 to 100 subclinical cases which are not reported due to the benign clinical manifestations and the lack of need for hospitalization (Christodoulou et al., 2012; Gradoni, 2013). In Greece, the mean annual incidence for the period 1998–2011 was 0.36/100,000 (Gkolfinopoulou et al., 2013). As for dogs, CanL which is endemic in more than 70 countries worldwide (Solano-Gallego et al., 2011), seroprevalence ranges between 2 and 37% depending on the region and the methods used for detecting the infection (Solano-Gallego et al., 2009; Athanasiou et al., 2012). With the more extensive use of molecular methods for the detection of Leishmanial DNA in canine tissues, has been shown a prevalence of infection between 67 to80% in some foci (Solano-Gallego et al., 2001; Leontides et al., 2002; Moreno and Alvar, 2002).

## Wild and synanthropic reservoir hosts of *Leishmania* spp. in nature

A possible definition for a reservoir host of an infectious agent in nature, is the ecological system where this agent can be maintained permanently, which system, in the case of a vector-borne agent involves one or more vectors and one or more mammalian hosts. Thus, we can say that a reservoir is a mammalian host that is responsible for the long-term preservation of this agent in nature (Ashford, 1996; Haydon et al., 2002). In the case of *Leishmania* spp., apart from the primary reservoir host, in the same area it is possible for other animals to be infected incidentally- incidental hosts. Incidental hosts are not related to the long-term preservation of the parasite, but under circumstances these host may become secondary reservoir hosts and be the source of infection for the both the vectors and humans (Shaw, 1988; Rotureau, 2006). The distinction between a primary and a secondary reservoir host is not easy (Quinnell and Courtenay, 2009). For a mammal to be incriminated as a primary reservoir host of *Leishmania* spp. parasites, it must be shown that this mammal is necessary for the preservation of the parasite in nature, which requires extensive ecological studies. Practically, for an animal species to serve as a primary reservoir host, a number of criteria are set by the WHO(1984): Specifically, (1). Such species should have long lifespan and must be of a minimum population in a certain area to serve as a good source of blood in order sand fly vectors to get infected. (2). In a such population, a large proportion of animals should become infected, in some cases over 20% of the population, even though the prevalence of infection varies with season (WHO, 2010). (3). The animals of the population should also be exposed to infection for prolonged periods of time, without particularly showing severe signs of the disease, but should allow for the vectors to become infected though skin or blood (4). Sand flies should have an intense contact with the host animal to facilitate the transmission of the parasite, (5) The parasite species of the animal population should have the potential to infect humans (WHO, 1990). One of the most impressive achievements of the *Leishmania* spp. parasites is the fact that they successfully parasitize the host’s macrophages, which are in fact the cells that are responsible for killing invaders (Shaw, 1997). These extremely successful parasites infect several mammalian species that belong to several orders: Carnivora, Chiroptera, Cingulata, Hyracoidea, Marsupialia, Perissodactyla, Pilosa, Primata and, Rodentia, (Dantas-Torres, 2007; Roque and Jansen, 2014). Thus, infection with *L. donovani* has been reported in Sudan in Egyptian mongooses (*Herpestes ichneumon*) and in the rodent species *Mastomys natalensis* and *Arvicanthis niloticus* (Elnaiem et al., 2001) and in Ethiopia in the rodent species *Arvicanthis* spp., *Mastomys erythroleucus* and *Gerbilliscus nigricaudus* (Kassahun et al., 2015). Infected with *L.* tropica were reported in Ethiopia the rodent species *Gerbillus nanus* and *Acomys* spp. (Kassahun et al., 2015), in Egypt the rodent species *Gerbillus pyramidum floweri* (Shehata et al., 2009) and with *L. gerbilli* και *L. turanica* in great gerbils (*Rhombomys opimus)* in Iran (Akhavan et al., 2010). *L infαntum* infection has been reported in several rodent species (*Meriones persicus, Cricetalus migratorius, Mesocricetus auratus* etc.), foxes (*Vulpes vulpes*), jacals (*Canis aureus)* and wolves (*Canis lupus*) in Iran (Mohebali et al., 2005; Fallah et al., 2006), in foxes and jacals in Israel (Baneth et al., 1998), in Norwegian rats (*Rattus norvegicus)* in Brazil (Lara-Silva et al., 2014). Similarly, several rodent species (*R. rattus, Sigmodon hispidus*, *Thrichomys apereoides* etc.) were reported infected with *L. mexicana and L. braziliensis* in Venezuela (De Lima et al., 2002), Brazil (Oliveira et al., 2005; Marcelino et al., 2011) and Mexico (Canto-Lara et al., 1999).

## Reservoir hosts of *L. infantum* in Europe

**Fig. 1:**
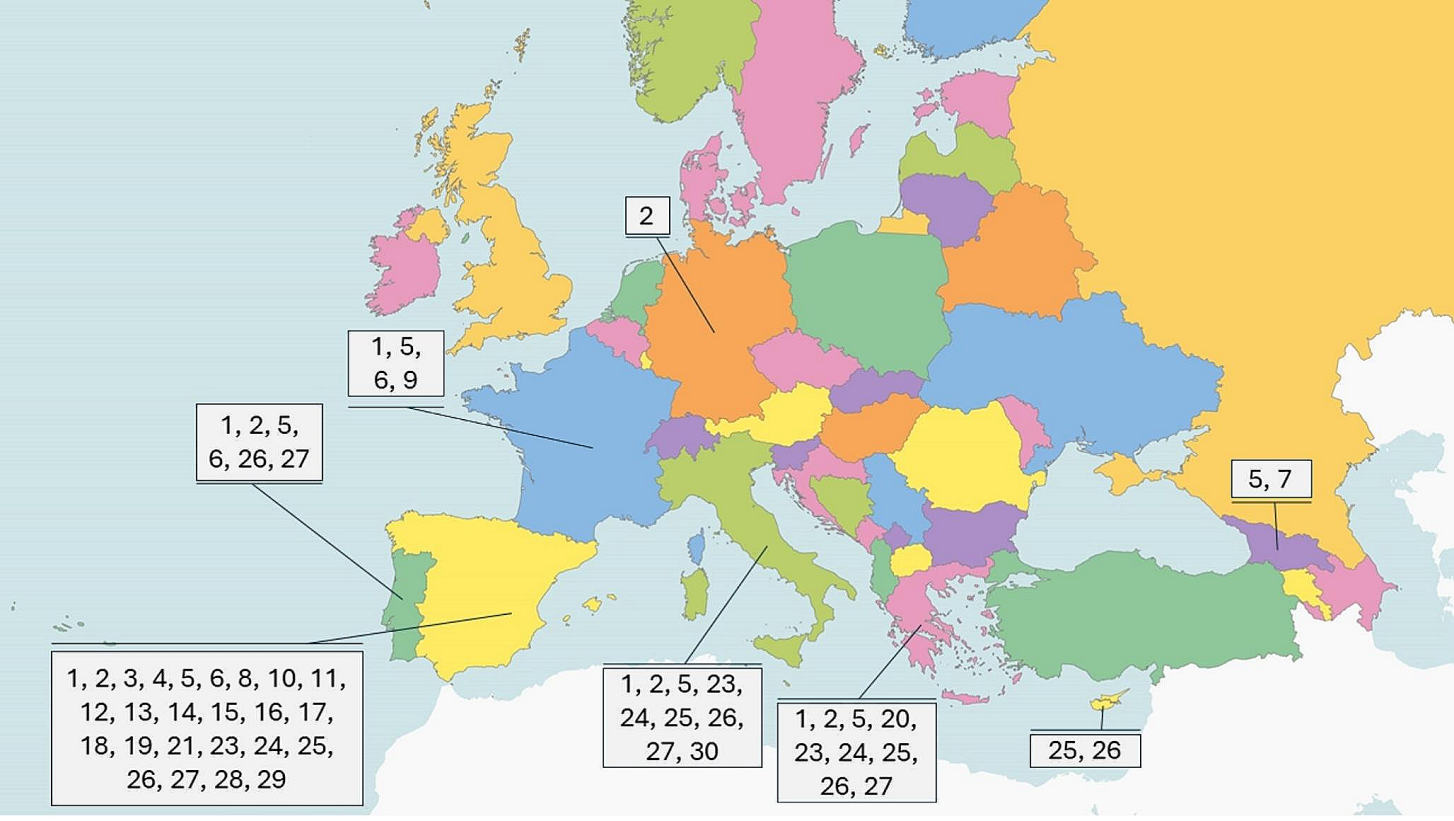
Host species of *Leishmania infantum* that have been identified in different European countries: (1) Cat, (2) Horse, (3) Sheep, (4) Goat, (5) Red fox, (6) Grey wolf, (7) Golden jackal, (8) European wildcat, (9) Barbary lion, (10) Tiger, 11. Eurasian otter, 12. Pole cat, 13. European pine marten, 14. Stone marten, 15. Common genet, 16. Iberian lynx, 17. Egyptian mongoose, 18. European badger, 19. European mink, 20. American mink, 21. Brown bear, 22. Common pipistrelle bat 23. Wild rabbit, 24. Hare, 25. Black rat, 26. Norwegian rat, 27. House mouse, 28. Algerian mouse, 29. Wood mouse, 30. Lizard CYPRUS: 25, 26; FRANCE: 1, 5, 6, 9; GEORGIA: 5, 7; GERMANY: 2; GREECE: 1, 2, 5, 20, 23, 24, 25,26, 27; ITALY: 1, 2, 5, 23, 24, 25, 26, 27, 30; PORTUGAL: 1, 2, 5, 6, 26, 27; SPAIN: 1, 2, 3,4, 5, 6, 8, 10, 11, 12, 13, 14, 15, 16, 17, 18, 19, 21, 23, 24, 25, 26, 27, 28, 29

In Europe, the domestic dog is the proven to be the main reservoir host for *L. infantum* parasites while other animal species can serve as potential hosts. Figure 1 illustrates different host species that have been identified in different European countries. However, the rising incidence of ZVL in endemic areas of the world, is an indication that the currently applied control measures focused on sand fly and dog control, with the latter based on culling of seropositive dogs, or treatment of dogs with permethrin and deltamethrin (Ferroglio et al., 2008), are not entirely effective (Quinnell and Courtenay, 2009; WHO, 2010; Millán et al., 2011). Apart from the problems associated with the implementation of dog culling, the failure of leishmaniosis control, when the measures were focused on the dogs, led to the assumption that there might be alternative reservoir hosts of the parasite and it is possible that a peri-domestic and sylvatic cycle, with different mammalian species as primary reservoirs, can exist concurrently (Sobrino et al., 2008; Maia and Campino, 2011). Various animal species, domestic and wild, have been recorded to be naturally infected with *L. infantum.* Infection in domestic animals was reported in cats, equids, sheep and goats. A summary of the studies conducted across European countries in different wild and domesticated species that serve as hosts on *L. infantum* is presented in Table 1.

**Table 1:** Findings of the reviewed sources

Hosts	Prevalence %	Detection method	Country	Reference
Domestic animals				
Cat	100.0	Isoenzyme Electrophoresis	France	Ozon et al. 1998
	3.9–41.0	ELISA, PCR	Greece	Diakou et al. 2009, Chatzis et al. 2014
	0.8–8.6	IFAT, PCR	Italy	Poli et al. 2002, Iatta et al. 2019, Spada et al. 2020
	0.3–30.4	DAT, ELISA, IFAT, PCR	Portugal	Maia et al. 2008, 2010, Cardoso et al. 2010, Duarte et al. 2010, Vilhena et al. 2013
	0.4–60.0	IFAT, PCR	Spain	Martín-Sánchez et al. 2007, Ayllon et al. 2008, Miró et al. 2014
Horse	100.0	PCR	Germany	Koehler et al. 2002
	0.3	ELISA	Greece	Kouam et al. 2010
	6.5	IFAT	Italy	Sgorbini et al. 2014
	7.7	CIE, PCR	Portugal	Rolào et al. 2005
	14.3–100.0	ELISA, LPA, Immunohistochemical staining	Spain	Solano-Gallego et al. 2003 Fernández-Bellon et al. 2006
Sheep	13.9	ELISA	Spain	Fisa et al. 1999
Goat	10.2	ELISA	Spain	Fisa et al. 1999
Wild animals				
Carnivores				
Red fox	9.0	PCR	France	Davoust et al. 2014
	0.03	rK39 rapid test	Georgia	Babuadze et al. 2014
	59.5	PCR	Greece	Karayiannis et al. 2015
	28.6–52.2	PCR	Italy	Verin et al. 2010, Abbate et al. 2019
	1.3–60.0	DAT, IFAT, PCR	Portugal	Semião-Santos et al. 1996, Cardoso et al. 2015
	14.1–29.0	PCR	Spain	Sobrino et al. 2008, Del Río et al. 2014
Grey wolf	6.0-20.5	IFAT, PCR	France, Portugal, Spain	Sastre et al. 2008, Sobrino et al. 2008
Golden jackal	0.03	rK39 rapid test	Georgia	Babuadze et al. 2014
European wildcat	25.0	PCR	Spain	Del Río et al. 2014
Barbary lion	20.0–25.0	IFAT, PCR	France	Libert et al. 2012
Tiger	45.0	PCR	Spain	Iatta et al. 2020
Eurasian otter	100.0	ELISA, PCR, Microscopy	Spain	Cantos-Barreda et al. 2020
Pole cat	25.0	PCR	Spain	Del Río et al. 2014
European pine marten	30.0–39.0	PCR	Spain	Millán et al. 2011, Del Río et al. 2014
Stone marten	29.0	PCR	Spain	Del Río et al. 2014
Common genet	10.0–100.0	PCR	Spain	Sobrino et al. 2008, Millán et al. 2011 Del Río et al. 2014, Ortuño et al. 2019
Iberian lynx	25.0	PCR	Spain	Sobrino et al. 2008
Egyptian mongoose	28.6	PCR	Spain	Sobrino et al. 2008
European badger	26.0	PCR	Spain	Del Río et al. 2014
European mink	50.0	PCR	Spain	Del Río et al. 2014
American mink	2.1–20.0	ELISA, PCR	Greece	Tsakmakidis et al. 2019
Brown bear	100.0	PCR	Spain	Ortuño et al. 2019
Chiroptera				
common pipistrelle bat	59.3	PCR		Azami-Conesa et al. 2020
Lagomorphs				
Wild rabbit	4.2	PCR	Italy	Abbate et al. 2019
	1.0	ELISA, PCR	Greece	Tsakmakidis et al. 2019
	0.0–75.4	IFAT, PCR, rK39 rapid test	Spain	Chitimia et al. 2011, García et al. 2014 Jiménez et al. 2014, Moreno et al. 2014
Hare	9.8	PCR	Italy	Rocchigiani et al. 2018
	3.6–23.5	ELISA, PCR	Greece	Tsokana et al. 2015, Tsakmakidis et al. 2019
	43.6–74.1	IFAT, PCR	Spain	Ruiz-Fons et a. 2013, Moreno et al. 2014
Rodents				
Black rat	11.2	IFAT	Cyprus	Psaroulaki et al. 2010
	25.0–50.0	ELISA, PCR	Greece	Tsakmakidis et al. 2017
	15.5–57.5	IFAT, PCR	Italy	Di Bella et al. 2003, Zanet et al. 2014
	33.3	PCR	Spain	Navea-Pérez et al. 2015
Norwegian rat	5.6	IFAT	Cyprus	Psaroulaki et al. 2010
	6.3–70.0	ELISA, PCR	Greece	Papadogiannakis et al.2010, Tsakmakidis et al. 2017
	33.0	IFAT,	Italy	Di Bella et al. 2003
	33.3	Parasitological analysis	Portugal	Helhazar et al. 2013
	40.0–100.0	PCR	Spain	Ortuño et al. 2019
House mouse	24.0–50.0	ELISA, PCR	Greece	Tsakmakidis et al. 2017
	7.7	IFAT	Italy	Di Bella et al. 2003
	33.3	PCR	Portugal	Helhazar et al. 2013
	50.0	PCR	Spain	Navea-Pérez et al. 2015
Wood mouse	20.8	PCR	Spain	Navea-Pérez et al. 2015
Algerian mouse	42.9	ELISA	Spain	Alcover et al. 2020
Squamata reptiles				
Lizard	3.1	PCR	Italy	Mendoza-Roldan et al. 2022

### Domestic animals

#### Cats

Over the last century, domestic cats (*Felis catus*) were generally considered as unusual hosts for *Leishmania* spp. parasites, with a relatively high natural resistance to infection, possibly due to genetic factors and not strictly related to cell mediated immunity (Mancianti, 2004) and though these animals live in the same habitat with dogs and humans, only sporadic clinical cases of feline leishmaniosis had been reported (Tabar et al., 2008). Recently, advances in feline medicine and developments of sensitive diagnostic tools in serology and molecular analyses, showed that reported clinical and asymptomatic cases of leishmaniosis in cats were underestimated, nevertheless the detected prevalence of leishmaniosis in cats seems to be lower than in dogs as studies indicate in the same areas (Diakou et al., 2009; Maia et al., 2010; Penisi et al., 2015). As a result, there are a few surveys, based on serology and/or PCR that show infection in several European countries, were CanL and HumL are endemic, with prevalence ranging between 0.3 and 60% (Martín-Sánchez et al., 2007; Ayllon et al., 2008; Cardoso et al., 2010; Maia and Campino, 2011; Vilhena et al., 2013; Chatzis et al., 2014). Infected cats were reported in Portugal (Maia et al., 2008), Spain (Miró et al., 2014), Italy (Poli et al., 2002; Iatta et al., 2019), France (Ozon et al., 1998) and Greece (Diakou et al., 2009). The recent results on the susceptibility and the role of cats in the epidemiology of *L. infantum* in endemic areas remain controversial. There are studies which point out that cats appear to have natural resistance to infection (Diakou et al., 2009), are rare hosts of the parasite and consequently are not a serious threat for public health (Duarte et al., 2010; Miró et al., 2014), or can act as a secondary reservoir host which cannot maintain infection in an area unless there are also present infected dogs (Penisi et al., 2015). On the other hand, there are studies suggesting that cats are susceptible to the parasite, infectious to sand flies and thus, can have a role not as an accidental host but as an alternative reservoir host of the parasite (Maroli et al., 2007; Martín-Sánchez et al., 2007; Maia et al., 2010; Chatzis et al., 2014; Pennisi and Persichetti, 2018) In any case, the role of cats in the maintenance and transmission of the parasite needs to be further investigated (Spada et al., 2020; Cardoso et al., 2021).

#### Equids

*Leishmania* spp. infection in horses, is not uncommon in areas of South and Central America, with *L. braziliensis* being the identified species (Koehler et al., 2002; Madeira et al., 2006), where it was proposed that horses can acts as reservoirs in peri-urban areas of Brazil (Rolao et al., 2005). In Europe, equine leishmaniosis caused by *L. infantum*, has been reported in Spain, Portugal, Germany, Italy and Greece, with cutaneous leishmaniosis being the only clinical form described and seroprevalence ranging between 0.3% to14% (Koehler et al., 2002; Solano-Gallego et al., 2003; Rolao et al., 2005; Kouam et al., 2010; Sgorbini et al., 2014). Equids are incidental hosts of the parasite as evidenced by the the rare reported clinical cases, the low rate of infection (Kouam et al., 2010) and the spontaneous healing of the lesions (Koehler et al., 2002). All of these indicate that the immune response of these animals can prevent the progressing of disease (Fernández-Bellón et al., 2006). Nevertheless, leishmaniosis in equids, requires further investigation to clarify the clinical form of infection and the role of these animals in the epidemiology of the disease (Solano-Gallego et al., 2003; Rolao et al., 2005).

#### Sheep and goats

*Leishmania* spp. infection in sheep and goats is uncommon and only a few cases have been reported worldwide, as it was described in a sheep in Eastern Transvaal (*Leishmania* spp.), in a goat in Kenya (most likely *L. aethiopica*), in goats in Sudan (*L. donovani*) and in sheep and goats in China (*L. infantum*) (Williams et al., 1991; Van der Lugt et al., 1992; Mukhtar et al., 2000; Gao et al., 2015). In Europe, infection of sheep and goats was reported in Spain, with seroprevalence of 13.9% and 10.2% respectively (Fisa et al., 1999). As these farm animals share the same bio habitat with humans, their role in the epidemiology of the disease needs to by further investigated and elucidated.

### Wild animals

Natural infection in wild animals was reported in various animal species which belonged to the orders of carnivores, chiroptera, lagomorphs, rodents, and squamata reptiles.

#### Carnivores

Out of carnivores, the species that was the most extensively studied is the red fox (*Vulpes vulpes*) and a possible reason is that both foxes and dogs are canids, which means that they belong the same family of classification (Canidae) and moreover the red fox is the wild carnivore that is in greater numbers in the European continent (Millán et al., 2014). Red foxes were found positive for *Leishmania* spp. infection in Spain (Criado-Fornelio et al., 2000; Sobrino et al., 2008), Italy (Verin et al., 2010 ), France (Davoust et al., 2014), Portugal (Semião-Santos et al., 1996; Cardoso, et al., 2015), Georgia (Babuadze et al., 2014) and Greece (Karayiannis et al., 2015), with seroprevalence up to 60% amongst the aforementioned countries. Similarly, molecular analyses indicated a prevalence of infection up to 74.6% (Millán et al., 2014; Piantedosi et al., 2016; Abbate et al., 2019).

Leishmaniosis has also been reported in grey wolves (*Canis lupus*), golden jackals (*Canis aureus*), European wildcats (*Felis silvestris silvestris*), barbary lions (*Panthera leo*), tigers (*Panthera tigris*), Eurasian otters (*Lutra lutra*), pole cats (*Mustela putorius*), European pine martens (*Martes martes*), stone martens (*Martes foina*), common genets (*Genetta genetta*), Iberian lynxes (*Lynx pardinus*), Egyptian mongooses (*Herpestes ichneumon*), European badgers (*Meles meles*), European minks (*Mustela lutreola*), American minks (*Neovison vison*) and in brown bear (Ursus arctos) (Sastre et al., 2008; Millán et al., 2011; Libert et al., 2012; Babuadze et al. 2014; Del Río et al., 2014; Ortuño et al., 2019; Tsakmakidis, et al., 2019; Iatta et al., 2020; Cantos-Barreda et al., 2020). In most of the studies conducted on red foxes, most of the researchers postulate the notion that red foxes could be considered as wild reservoir hosts for *Leishmania* spp. (Criado-Fornelio et al., 2000; Dipineto et al., 2007; Davoust et al., 2014; Karayiannis et al., 2015). Red foxes live near urban and agricultural areas and are frequently exposed to sand flies. Moreover, studies in Europe showed a preference of *Phlebotomus pernicious* sand flies to them for their blood meal (Veronesi et al., 2023). Further studies are necessary to assess the role of red foxes and other wild carnivores in the epidemiology of the disease (Sobrino et al., 2008; Cardoso et al., 2021) and therefore the existence of other reservoir hosts, apart from dogs, should not be underestimated (Millán et al., 2011).

#### Chiroptera

Infection has been recorded in common pipistrelle bats (*Pipistrellus pipistrellus*) in Spain, when 59.3% were found infected with *L. infantum*, performing a molecular method (Azami-Conesa, et al., 2020).

#### Lagomorphs

The lagomorphs that were reported with infection were the wild rabbit (*Oryctolagus cuniculus*) and the hare species *Lepus europaeus*, *Lepus granatensis* and *Lepus castroviejoi* (Ruiz-Fons et al., 2013; Díaz-Sáez et al., 2014). Infected wild rabbits were found in Spain, Italy and Greece, with a seroprevalence up to 75.4%. Molecular analyses indicated prevalence of infection up to 20.7% (Chitimia et al., 2011; García et al., 2014; Jiménez et al., 2014; Moreno et al., 2014; Abbate et al., 2019; Tsakmakidis, et al., 2019). Infection in hares was reported in Spain, Italy and Greece, with rates reaching 74.1% when serology was used for diagnosis and 43.6% when a molecular method was used (Ruiz-Fons et al., 2013; Moreno et al., 2014; Tsokana et al., 2015; Rocchigiani et al., 2018; Tsakmakidis, et al., 2019).

Research studies on the role of these species implicated, for the first time, the hares as reservoirs in the epidemiology of leishmaniosis and more specifically, in the human leishmaniosis outbreak reported in the area of Madrid, Spain, during July 2009 and December 2012 (Molina et al., 2012; Acre et al., 2013). The high rates of infection reported in hares, combined with their proven ability, under xenodiagnostic studies, to infect sandflies, the fact that they are apparently healthy while infected and thus can sustain a chronic infection and the fact that they inhabit large areas in European continent, makes them a potential wild reservoir for *Leishmania* spp. in Europe (Molina et al., 2012; Ruiz-Fons et al., 2013; Jiménez et al., 2014). In rabbits on the other hand, results seem to be controversial, were there are studies suggesting that rabbits cannot possibly play the role as reservoirs of *L. infantum* (Chitimia et al., 2011), were others that rabbits show some of the characteristics expected in a possible reservoir host, as, like the hares they are long lived, at least they survive through one non-transmission period, show no apparent signs of an acute disease (Díaz-Sáez et al., 2014) and can be infective to sandflies, as it was proven with xenodiagnostic studies (Jiménez et al., 2014). In conclusion, most of the researchers agree that the impact of lagomorphs in the epidemiology of leishmaniosis in Europe needs to be further investigated.

#### Rodents

Infection of wild rodents was reported in black rats (*Rattus rattus)*, Norwegian rats (*Rattus norvegicus)*, house mice (*Mus musculus*), wood mice (*Apodemus sylvaticus*) and Algerian mice (*Mus spretus*). Infected black rats were reported in Italy, Spain, Cyprus and Greece, with a seroprevalence up to 57.5%, while molecular analysis indicated a rate of infection up to45% (Di Bella et al., 2003; Psaroulaki et al., 2010; Zanet et al., 2014; Navea-Pérez et al., 2015; Tsakmakidis et al., 2017). Infection of the Norwegian rat has been reported in Italy, Spain, Portugal, Cyprus and Greece with a seroprevalence up to 70%. In a molecular study conducted in Greece, the prevalence of infection was found to be 6.25% while in a parasitological study conducted in Portugal the prevalence of infection was determined as33.33% (Papadogiannakis et al., 2010; Psaroulaki et al., 2010; Helhazar et al., 2013; Tsakmakidis et al., 2017; Ortuño et al., 2019). House mice were found positive to infection with prevalence up to 50% as indicated in studies conducted in Italy, Portugal, Spain and Greece (Di Bella et al. 2003; Helhazar et al., 2013; Navea-Pérez et al., 2015; Tsakmakidis et al., 2017). Infected wood and Algerian mice were recorded in Spain when serology showed rates of infection 42.85% for Algerian mice and in in molecular studies the rate of infection for the wood mice was 20.8% (Navea-Pérez et al., 2015; Alcover et al., 2020). Most of the researchers agree to the fact that rodents show some of the characteristics of a reservoir host and possibly can have a role in the preservation of the parasite in nature and that it is necessary that research studies should be continued on the field of xenodiagnosis in order to evaluate the ability of these animals to infect the sand fly vectors (Helhazar et al., 2013; Zanet et al., 2014; Navea-Pérez et al., 2015). It must be noted here that high infection rates alone do not ensure that these animals can act as a reservoir host and detection of the parasite’s DNA in the examined tissues does not show conclusively active infection or the ability of the animal to infect sandflies, therefore xenodiagnostic studies are necessary to be applied (Del Río et al., 2014). So far, the ability to infect the sand fly vectors has already been confirmed by xenodiagnostic studies in domestic cats, hares, rabbits and black rats, a fact that increases for these animal species the possibility to act as reservoir hosts (Quinnell and Courtenay, 2009; Molina et al., 2012; Díaz-Sáez et al., 2014; Jiménez et al., 2014).

#### Squamata reptiles

Confirmed for *L*. *infantum* infection were identified by a molecular method 3,1% of examined lizards in Italy (Mendoza-Roldan et al., 2022).

## Conclusions

An emerging infectious disease can be described as a previously known or recently recognized infectious disease that showed a recent and fast increase in occurrence, geographic range, or recently moved into new host populations (Morse, 1995; Daszak et al., 2000). Under that definition are included several diseases that pose a serious threat to public health and a burden on global economies. This emergence is mostly resulting from socio-economic, environmental and ecological changes (CDC, 1998; Jones et al., 2008). The main event in most cases of emergence is a change in ecology between the host and the infectious agent. Wild animals are considered an important feature of this emergence, because in many cases are the reservoirs from which zoonotic pathogens can emerge (Daszak et al. 2001).

Human leishmaniosis due to *L. infantum* infection, is an important emerging zoonosis that shows an increasing frequency and a greater geographic distribution than the previous decades and although the domestic dog is the animal of the greatest importance and the main reservoir host in nature, there are several animal species, domestic and wild that are susceptible to the parasite’s infection (Maia et al., 2015). Even though there are a few studies in which the presence of *L. infantum* in various animal species has been demonstrated, the importance of these species as reservoirs remains largely unclear (Souza et al., 2014). This role of potential alternative host needs to be further explored, because, as it has been shown in previous cases, under certain circumstances can change and lead to serious public health problems, as it happened in the outbreak of leishmaniosis in Madrid (Arce et al., 2013). Epidemiology of the parasite is more complicated than we believed in the past (Navea-Pérez et al., 2015) and assessment of the possibility of other animals participating in Leishmania’s life circle is important.

## Data Availability

This is a review article and no experimental data were generated or analysed during the study. The bibliography based on which this manuscript was prepared on, is listed at the end of the manuscript.
